# New and continuing physician-based outpatient mental health care among children and adolescents during the COVID-19 pandemic in Ontario, Canada: a population-based study

**DOI:** 10.3389/fpsyt.2023.1063203

**Published:** 2023-11-06

**Authors:** Alene Toulany, Simone Vigod, Paul Kurdyak, Therese A. Stukel, Rachel Strauss, Longdi Fu, Astrid Guttmann, Jun Guan, Eyal Cohen, Maria Chiu, Charlotte Moore Hepburn, Kimberly Moran, William Gardner, Mario Cappelli, Purnima Sundar, Natasha Saunders

**Affiliations:** ^1^The Hospital for Sick Children, Toronto, ON, Canada; ^2^Department of Pediatrics, Temerty Faculty of Medicine, University of Toronto, Toronto, ON, Canada; ^3^ICES, Toronto, ON, Canada; ^4^Child Health Evaluative Sciences, SickKids Research Institute, Toronto, ON, Canada; ^5^Institute of Health Policy, Management and Evaluation, University of Toronto, Toronto, ON, Canada; ^6^Edwin S.H. Leong Centre for Healthy Children, University of Toronto, Toronto, ON, Canada; ^7^Department of Psychiatry, Temerty Faculty of Medicine, University of Toronto, Toronto, ON, Canada; ^8^Women’s College Hospital, Women’s College Research Institute, Toronto, ON, Canada; ^9^Centre for Addiction and Mental Health, Toronto, ON, Canada; ^10^Children’s Mental Health Ontario, Toronto, ON, Canada; ^11^Children’s Hospital of Eastern Ontario Research Institute, Ottawa, ON, Canada; ^12^School of Epidemiology and Public Health, University of Ottawa, Ottawa, ON, Canada; ^13^Knowledge Institute on Child and Youth Mental Health and Addictions, Ottawa, ON, Canada

**Keywords:** psychiatry, continuity of care, coronavirus, children, adolescents, mental health

## Abstract

**Objective:**

To assess physician-based mental health care utilization during the COVID-19 pandemic among children and adolescents new to care and those already engaged with mental health services, and to evaluate differences by sociodemographic factors.

**Study design:**

We performed a population-based repeated cross-sectional study using linked health and administrative databases in Ontario, Canada among all children and adolescents 3–17 years. We examined outpatient visit rates per 1,000 population for mental health concerns for those new to care (no physician-based mental healthcare for ≥1 year) and those with continuing care needs (any physician-based mental healthcare <1 year) following onset of the pandemic.

**Results:**

Among ~2.5 million children and adolescents (48.7% female, mean age 10.1 ± 4.3 years), expected monthly mental health outpatient visits were 1.5/1,000 for those new to mental health care and 5.4/1,000 for those already engaged in care. Following onset of the pandemic, visit rates for both groups were above expected [adjusted rate ratio (aRR) 1.22, 95% CI 1.17, 1.27; aRR 1.10, 95% CI 1.07, 1.12] for new and continuing care, respectively. The greatest increase above expected was among females (new: aRR 1.33, 95% CI 1.25, 1.42; continuing: aRR 1.22 95% CI 1.17, 1.26) and adolescents ages 13–17 years (new: aRR 1.31, 95% CI 1.27, 1.34; continuing: aRR 1.15 95% CI 1.13, 1.17). Mood and anxiety concerns were prominent among those new to care.

**Conclusion:**

In the 18 months following onset of the pandemic, outpatient mental health care utilization increased for those with new and continuing care needs, especially among females and adolescents.

## Introduction

The COVID-19 pandemic has created a unique set of risk factors for the development of new mental health disorders and exacerbation of pre-existing illness among children and adolescents. Concerns for increasing psychological distress and mental health disorders among children and adolescents emerged early in the COVID-19 pandemic, as school closures and physical distancing orders isolated young people from their peers and significantly disrupted existing routines (1, 2). In Canada and other jurisdictions, many children and adolescents have experienced significant increase in emotional distress, changes in mood, symptoms of anxiety and depression, and this has translated into greater utilization of mental health services (3–6). However, as more research has materialized during the pandemic, it is evident that study findings on the magnitude of the mental health impacts on young people are conflicting (7). While many studies have reported negative mental health outcomes for children and adolescents during the pandemic such as increases in depression, anxiety, and eating disorders (4, 5, 8, 9), others have reported stable or declining rates of self-harm, suicidality, and substance use (4, 5, 8–11). Given the rapidly evolving nature of the pandemic and predicted long-term economic and social consequences, there is a need to better understand the impact on children and adolescents’ mental health over time. Likely, the short-and long-term effects on mental health will differ across age and sociodemographic groups (12, 13), with some being exceptionally vulnerable.

Given the major biologic and psychosocial developmental changes during childhood and adolescence, young people may be particularly vulnerable to pandemic-related stressors including loss of routines and supports, social isolation, lack of control, fear of infection and loss, and parental/caregiver stress (1, 2, 12, 14–17). However, the frequency with which children and adolescents have presented to care with acute mental health concerns during the pandemic has not matched the level of distress that has been reported in pediatric populations in the community (7, 18). For example, while pediatric emergency department visits and hospitalizations for mental health concerns decreased or remained near expected levels for the first year following the pandemic in many health jurisdictions (6, 19–23), mental health-related outpatient visits sustained a 10% to 15% increase above expected following the first few months of the pandemic onset (6).

One major limitation of the literature to date has been a failure to distinguish those who newly needed to seek mental health care from those who were already engaged in care. This distinction may help to explain some of the conflicting findings in the existing literature. Challenges resulting from the COVID-19 pandemic may have heightened the risk for new-onset disorders, and/or exacerbated mental health issues for children and adolescents with pre-existing mental health disorders, but the nature of the presentations and the extent of care and resources required might differ greatly. The issue of access to health care during the pandemic might have affected these two groups differently, with the massive shift in outpatient care from in-person to virtual and a likely general reluctance, particularly early in the pandemic, to visit hospital settings and/or reduced mental health services in acute care settings (6, 19). Further, help-seeking patterns related to new engagement in care may differ greatly from that in ongoing care, especially for marginalized groups facing barriers to care (1, 2, 11, 13, 24–28).

This study aimed to examine physician-based outpatient mental health care visits before and during the COVID-19 pandemic among children and adolescents with new and continuing mental health care needs, and to evaluate differences by sociodemographic factors. We hypothesized that mental health care utilization among children and adolescents would increase disproportionately for those new to mental health care, especially among adolescent females as these groups may be uniquely vulnerable to the pandemic stressors.

## Materials and methods

### Study design and population

This population-based repeated cross-sectional study including all children and adolescents ages 3 to 17 years living in Ontario, Canada and eligible for provincial health insurance using linked health administrative databases housed at ICES, an independent, non-profit research institute whose legal status under Ontario’s health information privacy law allows it to collect and analyze health care and demographic data, without consent, for health system evaluation and improvement. We identified all physician-based pediatric mental health-related visits before (January 1, 2017, to February 29, 2020) and during (March 1, 2020, to December 31, 2021) the COVID-19 pandemic for those new to mental health care and with continuing service needs. We excluded non-Ontario residents, individuals with invalid birth dates and deaths within the study period, and those with missing data on sex.

### Data sources

Health and demographic administrative data were accessed from several databases linked using unique ICES encoded identifiers. The Ontario Health Insurance Plan (OHIP) physician claims database for insured services was used to determine outpatient visits to family physicians, pediatricians, or psychiatrists for mental health care. We used the Registered Persons Database (RPDB) to capture sociodemographic variables including date of birth, sex, date of death (if applicable), and postal code of all Ontario residents eligible for the province’s publicly funded universal health coverage, Ontario Health Insurance Plan (OHIP). Mental health-related emergency department visits were identified using the National Ambulatory Care Reporting System (NACRS), and the Canadian Institute for Health Information’s Discharge Abstract Database (CIHI-DAD) and the Ontario Mental Health Reporting System (OMHRS) were used to identify for mental health-related hospitalizations. Immigrant and refugee status was ascertained from the Immigration, Refugees and Citizenship Canada (IRCC) Permanent Resident Database. The linkage rate of the IRCC to population registries has been found to be 86% in Ontario (29). The Ontario Marginalization Index (ON-Marg) for neighbourhood material deprivation combines census information on income and education and was used as a measure of socioeconomic status. Quintiles are used to define the marginalization index, with 1 representing the least deprived neighbourhoods and 5 representing the most deprived neighbourhoods. Urban/rural region of residence was determined by the Rurality Index of Ontario (RIO) score, which is a continuous and broader measure of rurality used for policy development purposes in Ontario based on census subdivision (CSD), linked with Statistics Canada Postal Code Conversion File (PCCF) from postal code to Canadian Census data. Scores on the RIO are on a 100-point scale, with rural residence defined as a score of ≥40.

### Outcome measures

The primary outcome was monthly outpatient mental health-related visits (e.g., mental health diagnoses and concerns) to a family physician, pediatrician or psychiatrist per 1,000 population (Supplementary Table S1). The numerator represents unique mental health-related visits, not individual children and adolescents. This outcome was chosen as it measures a concept that combines both patient need and demand for services as well as physician access (6, 8, 13, 30). During the pre (January 1, 2017, to February 29, 2020) and peri-COVID (March 1, 2020 to December 31, 2021) periods, we identified individuals with these visits to create two monthly rolling cohorts consisting of (1) those new to mental health care (defined as no prior mental health claims, acute or outpatient, within 1 year prior to the 1st mental health visit date in the given month), and (2) those with continuing mental health care needs (any mental health-related claim in the year prior to the 1st mental health visit date in the given month). For example, if an individual from the new to mental health care cohort had a mental health visit in a given month, then all visits for this patient during the month would count into the numerator of new visits. The continuing care cohort the represents the number of visits per 1,000 among those already in care, not how many children and adolescents already in care had a visit.

Visit rates were expressed per 1,000 population overall and by three social determinants of health: neighbourhood socioeconomic status as measured by material deprivation, urban/rural region of residence, and individual immigration and refugee status. We also stratified by age, sex, and nature of the mental health disorder using physician diagnostic codes (psychotic disorders, mood and anxiety disorders, substance use disorders, social problems, and neurodevelopmental and other concerns) based on validated mental health physician service billing codes (Supplementary Table S1) widely used for mental health system performance reporting in Ontario and Canada (31, 32).

### Statistical analysis

Monthly outpatient mental health care visit rates per 1,000 children and adolescents were calculated, denominated on the Ontario population aged 3–17 years of the corresponding study year. We used Poisson generalized estimating equations (GEE) models for clustered count data to measure monthly and overall changes in rates of outpatient mental health visits among those new to and continuing mental health care. We modeled 3 years pre-COVID trends and used these to predict the expected trends during the COVID period (the first 18 months of the COVID-19 pandemic, from March 1, 2020, to December 31, 2021, representing the period of complete data availability following the pandemic onset) in the absence of public health restrictions, separately for each stratum. The model included age group-sex indicators, a continuous linear term measured as months since January 1, 2017 to estimate any overall trend in pre-COVID visit rates, and pre-COVID month indicator variables to model monthly variations, with April as the reference month. An autoregressive correlation structure with a lag of 1, to account for correlations in visit rates over time was used. The dependent variable was the stratum-specific count of visits among the specific cohort (new or continuing) in the stratum; the offset was the log of the stratum-specific population. We applied the linear combination of regression coefficients from pre-COVID to calculate the expected log rates in the COVID period, and computed the ratio of observed to expected rates in the COVID period by exponentiating the difference between the observed and expected log rates and 95% confidence intervals (CI). In additional secondary analyses, visit rates were stratified by social determinants of health, age, sex, and mental health diagnostic grouping.

We quantified monthly rate ratios of observed compared with expected mental health visit rates through the peri-COVID period (March 1, 2020 through December 31, 2021), as well as by early-and peri-COVID-19 periods (March 2020–June 2020 and July 2020–December 2021), respectively, as the first 3 months of the pandemic exhibited large decreasing trends in mental health service use due to major system access disruptions.

All analyses were performed using SAS version 9.4 (SAS Institute, Cary, North Carolina, United States). The use of these data was authorized under section 45 of Ontario’s Personal Health Information Protection Act, which does not require review by a Research Ethics Board, and cell sizes <6 were suppressed to meet institutional policy. We used the Reporting of Studies Conducted Using Observational Routinely-Collected Data (RECORD) reporting guideline.

## Results

Sociodemographic characteristics were relatively stable over the 3 years baseline period. In 2021, there were 2,426,171 children and adolescents aged 3–17 years living in Ontario (Table 1), with a mean age of 10.1 years (SD 4.28). Almost half were female (48.7%, *n* = 1,181,156). The majority lived in urban centres (90.0%, *n* = 2,184,690) and were Canadian-born (89.8%, *n* = 2,179,132). Approximately one-third (34.9%, *n* = 848,697) were living in the most deprived neighbourhoods (i.e., quintiles 4 or 5).

**Table 1:** Baseline demographic characteristics of children and adolescents, ages 3 to 17 years in Ontario, 2017–2021.

Year	2017	2018	2019	2020	2021
Children and adolescents on January 1st, *N*	2,377,289	2,393,927	2,416,427	2,443,606	2,426,171
**Age**					
Mean ± SD	10.09 ± 4.31	10.08 ± 4.29	10.09 ± 4.29	10.10 ± 4.29	10.11 ± 4.28
Median (IQR)	10 (6–14)	10 (6–14)	10 (6–14)	10 (6–14)	10 (6–14)
**Age group *n* (%)**					
3 to 12 years	1,572,135 (66.1)	1,584,983 (66.2)	1,599,073 (66.2)	1,615,946 (66.1)	1,600,090 (66.0)
13 to 17 years	805,154 (33.9)	808,944 (33.8)	817,354 (33.8)	827,660 (33.9)	826,081 (34.0)
**Sex, *n* (%)**					
Female	1,157,190 (48.7)	1,165,478 (48.7)	1,176,762 (48.7)	1,189,716 (48.7)	1,181,156 (48.7)
Male	1,220,099 (51.3)	1,228,449 (51.3)	1,239,665 (51.3)	1,253,890 (51.3)	1,245,015 (51.3)
**Rurality, *n* (%)**					
Urban	2,139,111 (90.0)	2,154,930 (90.0)	2,175,685 (90.0)	2,200,403 (90.0)	2,184,690 (90.0)
Rural	232,836 (9.8)	233,905 (9.8)	235,667 (9.8)	238,287 (9.8)	236,935 (9.8)
Missing	5,342 (0.2)	5,092 (0.2)	5,075 (0.2)	4,916 (0.2)	4,546 (0.2)
**Immigration status, *n* (%)**					
Non-refugee immigrants	142,452 (6.0)	128,019 (5.3)	112,284 (4.6)	98,159 (4.0)	83,656 (3.4)
Refugees or other immigrants	40,334 (1.7)	37,269 (1.6)	33,584 (1.4)	29,984 (1.2)	26,343 (1.1)
Newcomers	0 (0.0)	25,569 (1.1)	67,395 (2.8)	115,871 (4.7)	137,040 (5.6)
Canadian-born	2,194,501 (92.3)	2,203,069 (92.0)	2,203,164 (91.2)	2,199,592 (90.0)	2,179,132 (89.8)
**Material deprivation quintile, *n* (%)**					
Q1 (lowest)	554,645 (23.3)	566,171 (23.7)	579,107 (24.0)	590,365 (24.2)	586,023 (24.2)
Q2	510,798 (21.5)	514,656 (21.5)	520,516 (21.5)	526,610 (21.6)	522,021 (21.5)
Q3	437,272 (18.4)	438,782 (18.3)	441,164 (18.3)	445,078 (18.2)	442,487 (18.2)
Q4	398,832 (16.8)	399,339 (16.7)	401,072 (16.6)	403,954 (16.5)	401,487 (16.5)
Q5 (highest)	448,685 (18.9)	447,904 (18.7)	447,166 (18.5)	450,165 (18.4)	447,210 (18.4)
Missing	27,057 (1.1)	27,075 (1.1)	27,402 (1.1)	27,434 (1.1)	26,943 (1.1)

SD, standard deviation; IQR, interquartile range; Q, quintile.

### Changes in new and continuing mental health care visits

**Figure 1:**
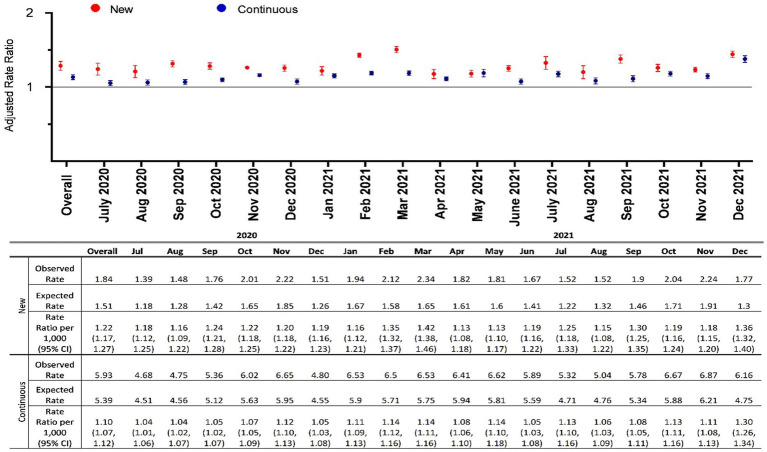
Adjusted monthly rate ratio of observed mental health-related visit rates following the onset of the COVID-19 pandemic compared to expected rates among children and adolescents with new and continuous care needs in Ontario.

**Figure 2:**
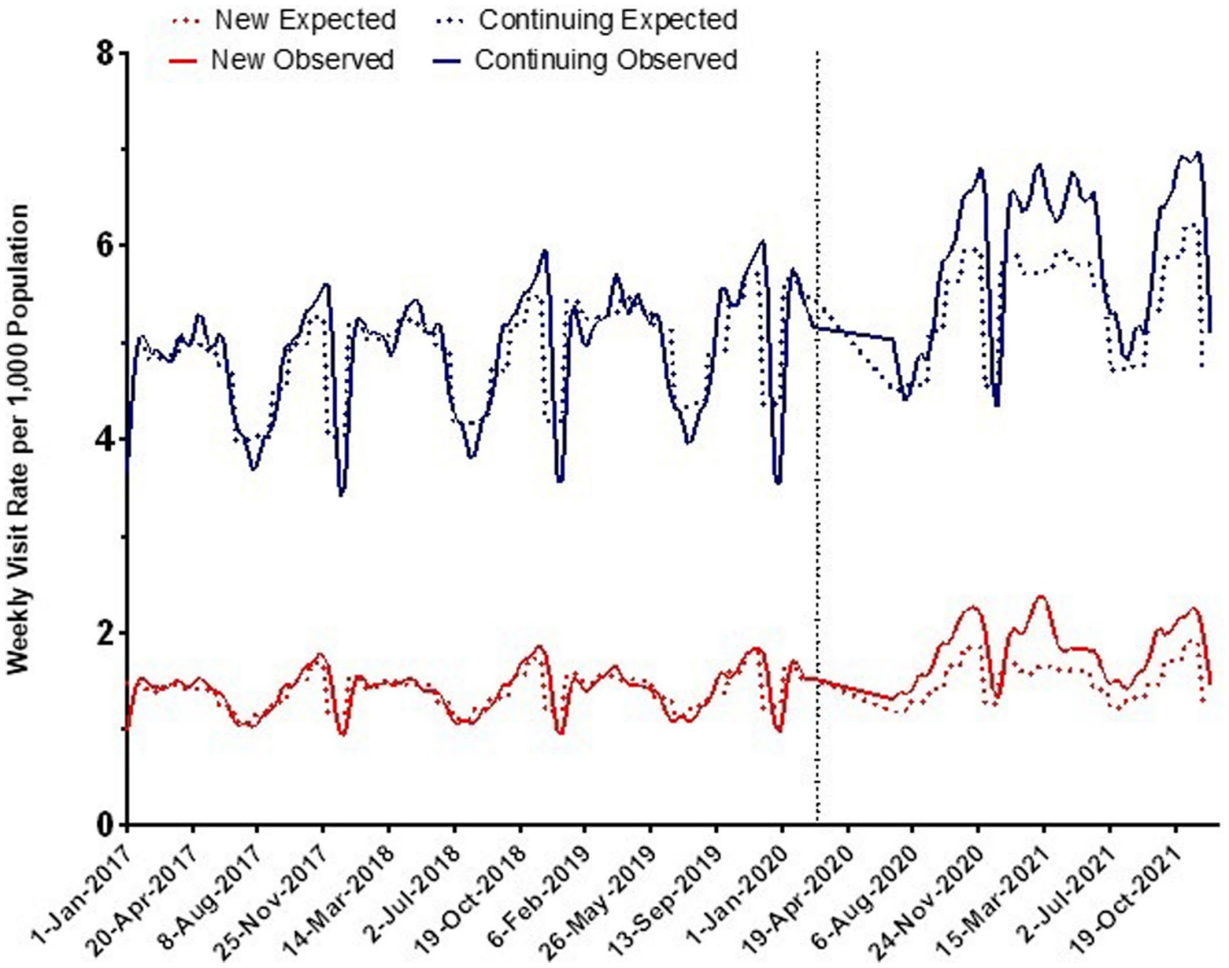
Observed and expected rates of outpatient mental health care visits over time in Ontario before and during the COVID-19 pandemic (per 1,000 population).

Overall, expected monthly rates of pediatric mental health outpatient visits during the pandemic were 1.5/1000 for those new to mental health care and 5.4/1,000 for those already engaged in care. Observed rates during the first 18 months of the pandemic increased above expected to 1.8/1,000 for those new to care and 5.9/1,000 for those already engaged with services (Figure 1). Overall, visits among those new to care increased by 22% [adjusted rate ratio (aRR) 1.22, 95% CI 1.17, 1.27] compared to expected rates, whereas those continuing care increased by 10% (aRR 1.10, 95% CI 1.07, 1.12). Initially following the pandemic onset, mental health visits decreased until July 2020, after which rates began to increase above expected (Figures 1, 2). Examining monthly trends, we observed an increase in visit rates consistently well above expected ranging from 18% in July 2020 (aRR 1.18, 95% CI 1.12, 1.25) and peaking to 42% in March 2021 (aRR 1.42, 95% CI 1.38, 1.46) for those new to mental health care (Figure 1). In contrast, we observed only a modest increase in mental health visit rates among those continuing to receive mental health care during the pandemic, typically ranging from 4% to 14% above expected levels.

### Sex and age

**Figure 3:**
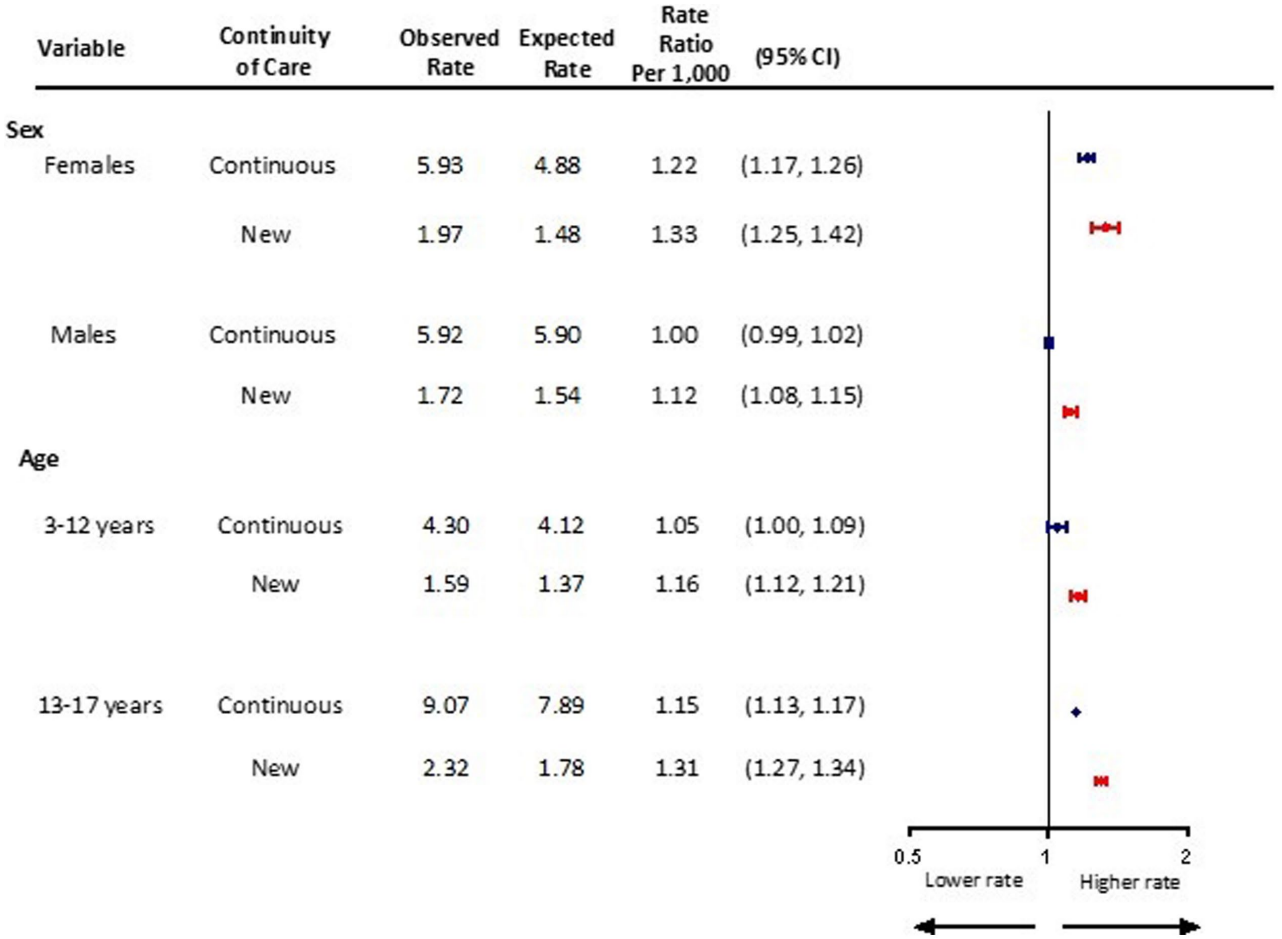
Adjusted monthly rate ratio of observed mental health-related visit rates following the onset of the COVID-19 pandemic compared to the expected rates based on 3 years pre-pandemic rates, by sex and age in Ontario.

Following the pandemic onset, females new to mental health care had the greatest overall increase in visit rates above expected (new: aRR 1.33, 95% CI 1.25, 1.42; continuing: aRR 1.22 95% CI 1.17, 1.26) (Figure 3). Visit rates among males new to mental health care were also above expected levels (aRR 1.12, 95% CI 1.08, 1.15), however, not for those continuing to receive care during the pandemic (aRR 1.00, 95% CI 0.99, 1.02). A 16% increase in visit rates above expected was observed among children ages 3–12 new to mental health care (aRR 1.16, 95% CI 1.12, 1.21), compared with a 5% increase among those continuing to receive care during the pandemic (aRR 1.05, 95% CI 1.00, 1.09) (Figure 3). Visits rates among adolescents new to mental health care during the pandemic increased 31% above expected (aRR 1.31, 95% CI 1.27, 1.34), compared with a 15% increase among those with continuing care needs (1.15 95% CI 1.13, 1.17).

### Social determinants of health

**Figure 4:**
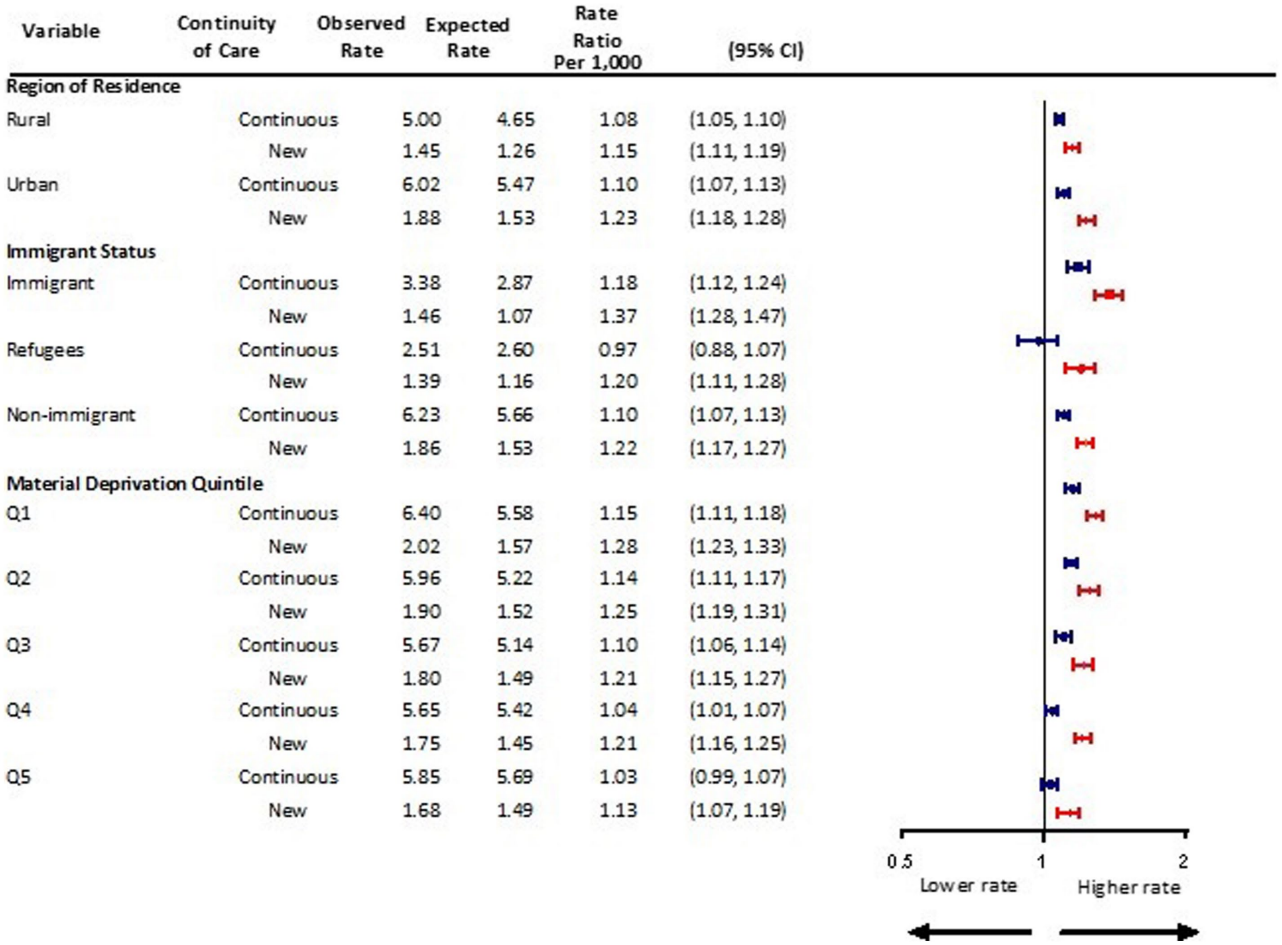
Adjusted monthly rate ratio of observed mental health-related visit rates following the onset of the COVID-19 pandemic compared to the expected rates based on 3 years pre-pandemic rates, by rurality, immigration status, and material deprivation in Ontario.

Following the onset of the pandemic, a 15% increase above expected was observed for children and adolescents new to mental health care in rural settings (aRR 1.15, 95% CI 1.11, 1.19), and a 23% increase in urban settings (aRR 1.23, 95% CI 1.18, 1.28) (Figure 4). For those with continuing mental health care needs, visit rates increased by 8% in rural (aRR 1.08, 95% CI 1.05, 1.10) and by 10% in urban regions of residence (aRR 1.10, 95% CI 1.07, 1.13).

Immigrant children and adolescents new to mental health care had a 37% increase in visit rates above expected (aRR 1.37, 95% 1.28, 1.47), whereas those with continuing care needs had an 18% increase (aRR 1.18, 95% 1.12, 1.24) (Figure 4). Refugees new to mental health care also had significantly higher than expected mental health visit rates during the pandemic (aRR 1.20, 95% CI 1.11, 1.28). This pattern was not seen among refugees with continuing mental health care needs (aRR 0.97, 95% 0.88, 1.07).

Similar trends were observed during the pandemic among children and adolescents new to mental health care across material deprivation quintiles with visit rates increasing by 13%–28% above expected (Figure 4). While mental health visits did not increase among children and adolescents with continuing mental care needs living in the most deprived neighborhoods (i.e., quintiles 5), such visits did increase for those residing in the least deprived areas.

### Mental health diagnostic groupings

**Figure 5:**
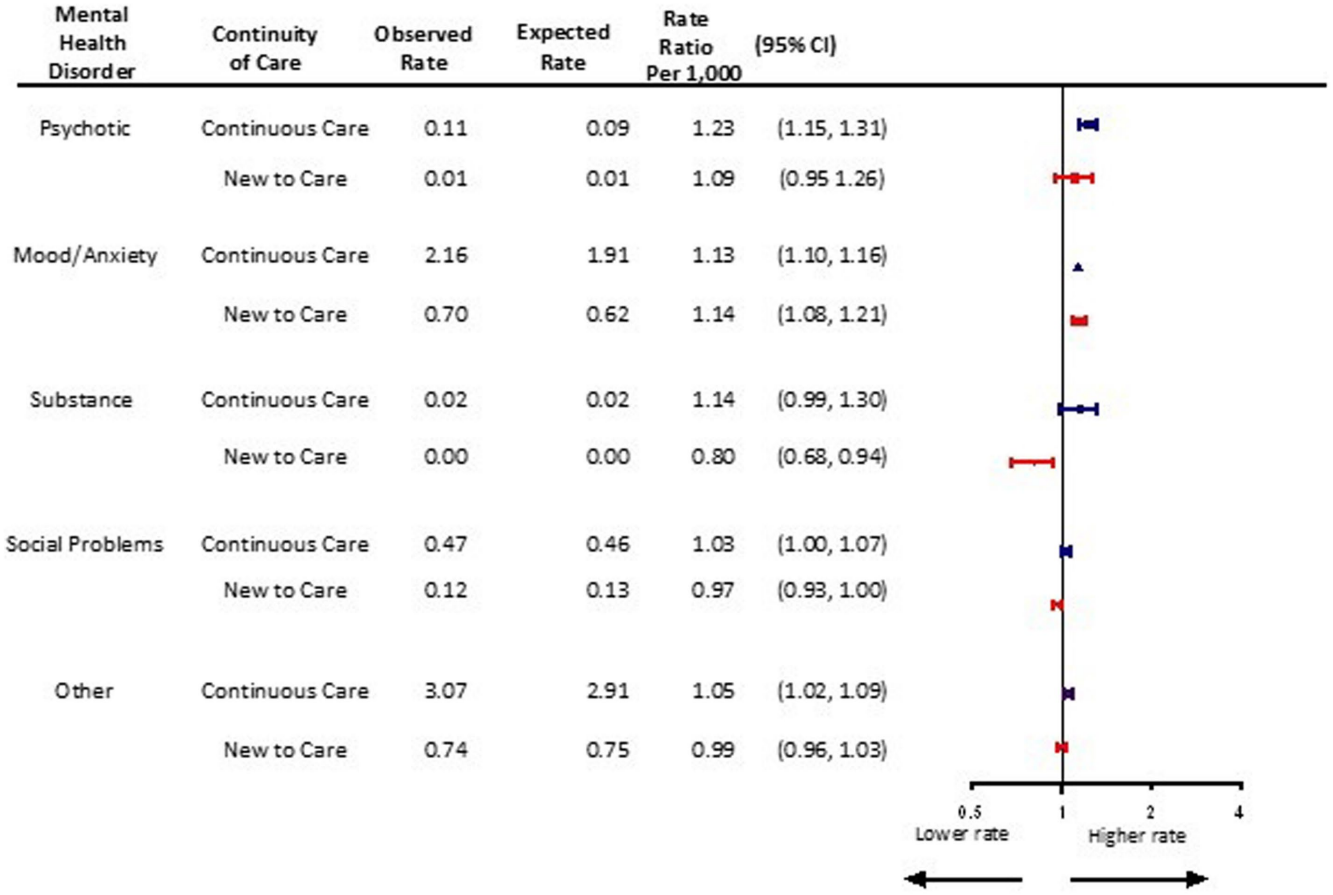
Adjusted monthly rate ratio of observed mental health-related visit rates following the onset of the COVID-19 pandemic compared to the expected rates based on 3 years pre-pandemic rates, by mental health disorder in Ontario.

Children and adolescents with both new and continuing care needs related to mood and anxiety disorders experienced greater than expected mental health visit rates following the pandemic (new: aRR 1.14, 95% CI 1.08, 1.21; continuing: aRR 1.13, 95% CI 1.10, 1.16) (Figure 5). In the first 18 months of the pandemic, observed monthly visit rates were 0.70/1,000 and 2.16/1,000 among those with new and continuing mental health care needs related to mood and anxiety disorders, compared to expected rates of 0.62/1,000 and 1.91/1,000, respectively. For psychotic disorders, observed monthly visit rates increased to 0.11/1,000, representing a 23% increase above an expected rate of 0.09/1,000 among those with continuing care needs (aRR 1.23, 95% CI 1.15, 1.31), but not for those new to care (aRR 1.09, 95% CI 0.95, 1.26). Visit rates for substance use disorders did not differ from expected levels in the 18 months following the pandemic onset (new aRR 0.80, 95% CI 0.68, 0.94 vs. continuing aRR 1.14, 95% CI 0.99, 1.30). Mental health visit rates for social problems and other mental health concerns were at or near expected levels.

## Discussion

In the 18 months following onset of the pandemic, physician-based outpatient mental health visit rates were 22% above expected based on pre-pandemic trends for children and adolescents with new-onset mental health concerns, and 10% above expected for those already engaged in mental health services. While the relative increase in visit rates were higher among those new to care, the absolute increases were higher among those with continuing care needs. Their overall monthly visit rates increased from 5.4/1,000 (expected) to 5.9/1,000 (observed), compared to an increase from 1.5/1,000 (expected) to 1.8/1,000 (observed) for those new to care. The greatest increases above expected were among females (34%), adolescents aged 13–17 years (31%) and children and adolescents of immigrant families (37%) new to mental health care. Mood and anxiety disorders were prominent diagnoses among both the new to care and continuing care groups with overall observed monthly visit rates of 0.70/1,000 and 2.16/1,000, respectively.

Findings from this population-based study fill an important gap in the literature on the changing characteristics of children and adolescents seeking outpatient mental health care during the pandemic and magnitude of the impact of cumulative stressors over time. The data signal that relatively more children and adolescents new to mental health care are seeking services to cope with their distress during the pandemic, while at the same time children and adolescents engaged with mental health services pre-pandemic continue to access care at slightly above expected rates. These findings are consistent with a US based study that reported a higher percentage of children and adolescents presenting for emergency psychiatric care during the pandemic with no prior mental health history (21), however, there were no differences observed in the types of new mental health disorders. Furthermore, a large cross-sectional Canadian study examining children and adolescents found a substantial increase in newly diagnosed anorexia nervosa with incidence increasing from 24.5 to 40.6 cases per month and hospitalizations among these patients increasing from 7.5 to 20.0 per month during the first wave of the COVID-19 pandemic (9).

The pandemic disrupted the most important aspects of children’s and adolescents’ development, especially in areas of skill achievement through school and play that are fundamental to their optimal growth, health, and emotional wellness (33–36). The distinction between new mental health presentations and continuing care is important to our evolving understanding of the pandemic’s impact on children and adolescents and the health care system receiving these patients. A critical difference is the additional comprehensive assessment that a new presentation usually entails, adding a substantial and relatively greater burden and cost on patients, their families, and the health care system as compared with those with continuing care needs. While the pandemic may have increased the need for new care or increased the need for more frequent care among those already engaged in care, the nature of need in these two groups may differ. For example, among those already in care, the increased need might translate into more intense care in the form of hospitalizations or emergency department visits. However, the dynamics of access to health care during the pandemic might affect the two groups differently, with the massive shift in outpatient care from in-person to virtual and a likely general reluctance, particularly early in the pandemic, to visit hospital settings and/or reduced services in acute care settings (6, 19). Understanding these differences and changing temporal patterns of both new and continuing mental health care visits during the pandemic is crucial as the scientific community, health system leaders, and decision makers apply research findings in real time to respond to the mental health needs of children and adolescents. Many health care systems, including that in Ontario, lack adequate capacity to manage the growing number of children and adolescents with mental health concerns, especially in the context of increasing demand on systems already stressed by the COVID-19 pandemic. While adequate capacity to meet the growing mental health service demands is important, ensuring continuity of care and clear pathways between primary care and community-based supports will be critical as the pandemic continues to evolve.

Published data remain limited on the changing characteristics of children and adolescents presenting to mental health care following onset of the pandemic. Our findings that female sex, adolescent age group, those living in the least deprived neighborhoods, and immigrants and refugees were disproportionately represented among new-onset mental health presentations following the pandemic builds on our previously published work and that of others (6, 13, 37, 38). The large sex-based and sociodemographic differences in physician-based mental health care visits suggests that the pandemic’s cumulative stressors may have had a disproportionately greater impact on adolescent females and certain disadvantaged populations, therefore, warranting closer monitoring and potential intervention. A report from the US Department of Health and Human Services/Centers for Disease Control and Prevention comparing data on mental-health related emergency department visits in 2019 and 2020 indicated that adolescents aged 12–17 years accounted for the largest proportion of visits following the pandemic, with rates increasing by 31% compared with 24% for younger children aged 5–11 years (28). An Ontario-based study of child and youth mental health assessments through multiple pandemic waves found an overall decline in the number of assessments across 53 select mental health agencies during the first wave of the pandemic, with some recovery during the second wave (38). Further, the authors report assessments of younger clients and males declined more than older clients and females. Other studies, however, have suggested little difference in sociodemographic characteristics during the pandemic apart from age (21, 39).

This study has several strengths. Our data encompasses a large population-based sample of almost all children and youth in Ontario, Canada. We utilized multiyear longitudinal pre-COVID data to predict health care utilization patterns 18 months following the pandemic onset. However, our results may not generalize to other health jurisdictions with different models of health care delivery, mental health services, and pandemic-related public health restrictions. Our administrative data are also limited by lags in data transfer, coding accuracy, and reflect mainly physician and hospital-based health care service use. Our data do not tell us the extent of unmet mental health care needs including those that are on waiting lists to be assessed. In addition, the use of neighbourhood-level socioeconomic variables in this study limits the extent to which we can make inferences about individual characteristics (e.g., being economically deprived is different than living in a geographic region that is economically deprived). While our findings signal a population-level increase in visits for new-onset mental health concerns among children and adolescents following onset of the pandemic, there are other equally important but unmeasured indicators not captured by our data that also signify serious distress. For example, many children and adolescents may access mental health supports from health care providers other than physicians including psychologists, social workers, and other therapists. Our databases do not capture services delivered by these other mental health professionals and therefore, we may have under reported the burden of new-onset illness and continuing service needs. Lastly, our data measures physician-based mental health care utilization through to December 2021 and future studies should examine whether and how patterns of new-onset mental illness presentations in children and adolescents following the pandemic change in the long-term.

The COVID-19 pandemic has created a distinct set of risk factors for the development of new mental health disorders and exacerbation of pre-existing illness among children and adolescents. We report significant differences in the number of pediatric patients presenting with new-onset mental health concerns compared with ongoing mental care needs during the pandemic relative to expected levels. Ongoing research is needed to better understand whether the increase in new-onset presentations will persist as the pandemic and recovery plans evolve. Furthermore, certain populations require close surveillance, particularly females, adolescents, marginalized populations and those with mood and anxiety disorders. Our findings have important implications for pandemic recovery planning as well as future pandemics, particularly expanding access to services and new pathways for mental health care across primary care, community, and hospital settings to better support children and adolescents with mental health service needs.

## Data availability statement

The original contributions presented in the study are included in the article/Supplementary material, further inquiries can be directed to the corresponding author.

## Author contributions

AT conceptualized and designed the study, interpreted the results, drafted the initial manuscript, and revised the manuscript. SV, PK, TS, RS, EC, AG, and NS conceptualized and designed the study, interpreted the results, and revised the manuscript. MCh, CH, KM, WG, MCa, and PS interpreted the results and revised the manuscript. LF and JG had access to and analyzed the data, interpreted the results and revised the manuscript. All authors contributed to the article and approved the submitted version.
