# Supplementary material for: New and continuing physician-based outpatient mental health care among children and adolescents during the COVID-19 pandemic in Ontario, Canada: a population-based study
Source: Front Psychiatry. 2023 Nov 6;14:1063203. doi: 10.3389/fpsyt.2023.1063203 (PMC10657868; doi:10.3389/fpsyt.2023.1063203)
Supplement: Supplementary file 1 [file Table_1.DOCX]

**Supplemental Files**

**eTable 1.** Ontario Health Insurance Plan and International Classification of Diseases, 8^th^ Revision mental health diagnostic codes and groupings.

| **Outpatient visit diagnostic codes, Ontario Health Insurance Plan** | | |
| --- | --- | --- |
| **Category** | **Diagnostic Code** | **Description** |
| **Psychotic Disorders** | 295 | Schizophrenia |
|  | 296 | Manic-depressive psychoses, involutional melancholia |
|  | 297 | Other paranoid states |
|  | 298 | Other psychoses |
| **Mood and anxiety Disorders** | 300 | Anxiety neurosis, hysteria, neurasthenia, obsessive-compulsive neurosis, reactive depression |
|  | 301 | Personality disorders |
|  | 302 | Sexual deviations |
|  | 306 | Psychosomatic illness |
|  | 309 | Adjustment reaction |
|  | 311 | Depressive disorder |
| **Substance Use Disorders** | 303 | Alcoholism |
|  | 304 | Drug dependence |
| **Social Problems** | 897 | Economic problems |
|  | 898 | Marital difficulties |
|  | 899 | Parent-child problems |
|  | 900 | Problems with aged parents or in-laws |
|  | 901 | Family disruption/divorce |
|  | 902 | Education problems |
|  | 904 | Social maladjustment |
|  | 905 | Occupational problems |
|  | 906 | Legal problems |
|  | 909 | Other problems of social adjustment |
| **Neuro-developmental and other concerns** | 291 | Alcoholic psychosis, delirium tremens, Korsakov's psychosis |
|  | 292 | Drug psychosis |
|  | 299 | Childhood psychoses (e.g., autism) |
|  | 307 | Habit spasms, tics, stuttering, tension headaches, anorexia nervosa, sleep disorders, |
|  | 313 | Behaviour disorders of childhood and adolescence |
|  | 314 | Hyperkinetic syndrome of childhood |
|  | 315 | Specified delays in development (e.g., dyslexia, dyslalia, motor retardation) |
| **Other mental health fee codes without diagnostic grouping** | **Fee code** | **Description** |
|  | K122 | Developmental and/or behavioural care - individual developmental and/or behavioural care |
|  | K123 | Developmental and/or behavioural care - family developmental and/or behavioural care |
|  | K704 | Paediatric outpatient case conference |
| **Virtual Codes** | Virtual visits were defined as those with either a location code indicating a virtual visit or that included any of the following virtual supplemental codes that were accompanied by an above mental health or addiction (MHA) diagnosis code (with the exception of K082 that does not require a MHA diagnosis code to be considered an MHA visit: B099, B100, B200, B101, B201, B102, B202, B103, B203, B209, K080, K081, K083 | |
